# Limitations of Transversus Abdominis Release (TAR)—Additional Bridging of the Posterior Layer And/Or Anterior Fascia Is the Preferred Solution in Our Clinical Routine If Primary Closure is Not Possible

**DOI:** 10.3389/jaws.2024.12780

**Published:** 2024-06-17

**Authors:** Hartwig Riediger, Ferdinand Köckerling

**Affiliations:** Vivantes Humboldt-Klinikum, Berlin, Germany

**Keywords:** incisional hernia, transversus abdominis release, robotic abdominal wall surgery, open surgery, bridging

## Abstract

**Background:** By separating the abdominal wall, transversus abdominis release (TAR) permits reconstruction of the abdominal wall and the placement of large mesh for many types of hernias. However, in borderline cases, the mobility of the layers is inadequate, and additional bridging techniques may be required for tension-free closure. We now present our own data in this regard.

**Patients and Methods:** In 2023, we performed transversus abdominis release on 50 patients as part of hernia repair. The procedures were carried out using open (n = 25), robotic (n = 24), and laparoscopic (n = 1) techniques. The hernia sac was always integrated into the anterior suture and, in the case of medial hernias, was used for linea alba reconstruction.

**Results:** For medial hernias, open TAR was performed in 22 cases. Additional posterior bridging was performed in 7 of these cases. The ratio of mesh size in the TAR plane to the defect area (median in cm) was 1200cm^2^/177 cm^2^ = 6.8 in patients without bridging, and 1750cm^2^/452 cm^2^ = 3.8 in those with bridging. The duration of surgery (median in min) was 139 and 222 min and the hospital stay was 6 and 10 days, respectively. Robotic TAR was performed predominantly for lateral and parastomal hernias. These procedures took a median of 143 and 242 min, and the hospital stay was 2 and 3 days, respectively. For robotic repair, posterior bridging was performed in 3 cases.

**Discussion:** Using the TAR technique, even complex hernias can be safely repaired. Additional posterior bridging provides a reliable separation of the posterior plane from the intestines. Therefore, the hernia sac is always available for anterior reconstruction of the linea alba. The technique can be implemented as an open or minimally invasive procedure.

## Background

The transversus abdominis release (TAR) technique first described in 2012 by Novitsky et al. is used predominantly to repair wide medial hernias (EHS W3, >10 cm) [1]. In 2016, a modified version of the technique was described by Pauli et al. to treat parastomal hernias [2]. The separation of the boundaries of the abdominal components permits the placement of meshes with a large overlap (TAR mesh) in the newly created extraperitoneal space. Furthermore, the separation of the abdominal wall into an anterior muscular plane and a posterior fascial plane increases the mobility of both layers. When properly performed, the technique ensures impressively good perioperative outcomes and rapid patient recovery [3, 4]. The recurrence rate is low, even in the long term [5, 6].

TAR can be performed using either open or minimally invasive surgery (MIS). For the latter, robotic surgery has become increasingly established. While the investment in equipment and instrumentation is considerable, the number of procedures carried out continues to grow. Based on our experience, one of the particular benefits of robotics is that lateral and parastomal hernias can also be repaired. If a mesh is to be placed in the extraperitoneal space, the anatomy of the abdominal wall in this location requires TAR regardless of the size of the hernia.

One of the difficulties that can be encountered when repairing complex abdominal hernias is the generation of excessive tension in the reconstruction phase. In the most unfavourable cases, this can even lead to life-threatening abdominal compartment syndrome. The TAR technique can recreate the physiologic abdominal volumes [7, 8]. However, if a plane cannot be closed, mesh-based anterior [8, 9], and much more commonly posterior [5, 10–18], bridging concepts have already been described for TAR. They are also firmly established in our clinical routine. Based on our own data from 2023, we can therefore present details of the surgical indications and the clinical treatment results of open and robotic repair with and without bridging.

We have been gaining practical experience with TAR for approximately 5 years now. Since the start of 2023 we have also had the privilege of using a DaVinci (Intuitive Surgical, Sunnyvale, CA, United States) system for hernia surgery. We have routine access to this 1 day a week. While the choice of procedure (open vs. robotic) is influenced by the different experience horizons, trends towards typical indications and concepts are emerging.

## Patients and Methods

In the period from January 01, 2023 to December 31, 2023, 88 medial and 15 lateral incisional hernias and 9 parastomal hernias were operated on in our hospital. Of these 112 patients, only those, (n = 50); (medial: n = 27, lateral: n = 14, parastomal: n = 9) who underwent open or minimally invasive transversus abdominis release with mesh placement were analysed in this study (Table 1). All patients were seen preoperatively by a qualified (FEBS AWS) surgeon and surgical repair by robotic or open technique was indicated. Due to the recent introduction of robotic hernia surgery last year, larger hernias were predominantly operated on using the open technique. Apart from our concept of TAR and optional bridging, no other hernia-specific reconstruction techniques were used. This was a retrospective study of prospectively collected data.

**TABLE 1:** Secondary hernias (n = 50) repaired in TAR technique in the period January 01, 2023 to December 31, 2023.

	Medial	Lateral	Parastomal
	(n = 27)	(n = 14)	(n = 9)
Open (n = 25)	22	1	2
Robotic (n = 24)	4	13	7
Laparoscopic (n = 1)	1	0	0

A particular focus of the study was the perioperative data of two TAR patient groups: the treatment outcomes of the clinically relevant group of patients with medial ventral hernia and open TAR repair were compared with respect to bridging (yes/no). The second analysis was for patients who underwent robotic repair of a medial, lateral or parastomal hernia. The open and robotic TAR surgical techniques have already been described in detail. We will therefore limit ourselves here to describing the technical aspects related to bridging. The surgical procedure was selected after individual assessment.

According to our clinical experience and a recent theoretical work-up in our group, patients with a defect width of 17 cm or more on the preoperative CT scan have a high probability of needing additional bridging [19]. We also expect posterior bridging in lateral defects of smaller dimensions when unilateral TAR is indicated to create a large extraperitoneal space for adequate mesh overlap (Figure 4).

### Steps in Open TAR Repair With Additional Bridging

The first step is to gain direct access to the abdominal cavity without developing the hernia sac from the subcutaneous layer. This means that the hernia sac will be available later for reconstruction of the linea alba. TAR is known to cause only limited medialisation in the anterior plane [20]. Deliberately leaving the hernia sac in place meant that we also had to rethink our views on the open concepts used up until now.

Next, adhesions are taken down from the anterior abdominal wall in all four quadrants. Interenteric adhesiolysis is only performed in patients with a history of impaired passage. The next step is to enter the retrorectus space.

It may be difficult to open the retrorectus space because the muscles are not only displaced laterally but are also compressed and narrowed. On CT, roll-shaped thickening of the rectus muscles is typically seen. After opening the medial edge, the posterior layer can be grasped with clamps and exposed. The TAR itself is then performed in a top-down or bottom-up technique parallel to the linea semilunaris with strict preservation of the neurovascular bundles that enter the retrorectus space 1–2 cm medial to the lateral margin. The extensions of the transversus abdominis (TA) reach cranially as far as the retrosternal fat tissue and merge with the diaphragm muscles. By detaching the muscles along the linea semilunaris, a common mesh plane is created from lateral to cranial and similarly in the lower abdomen, now extending from the retroxiphoid to the retro-symphyseal space.

**FIGURE 1:**
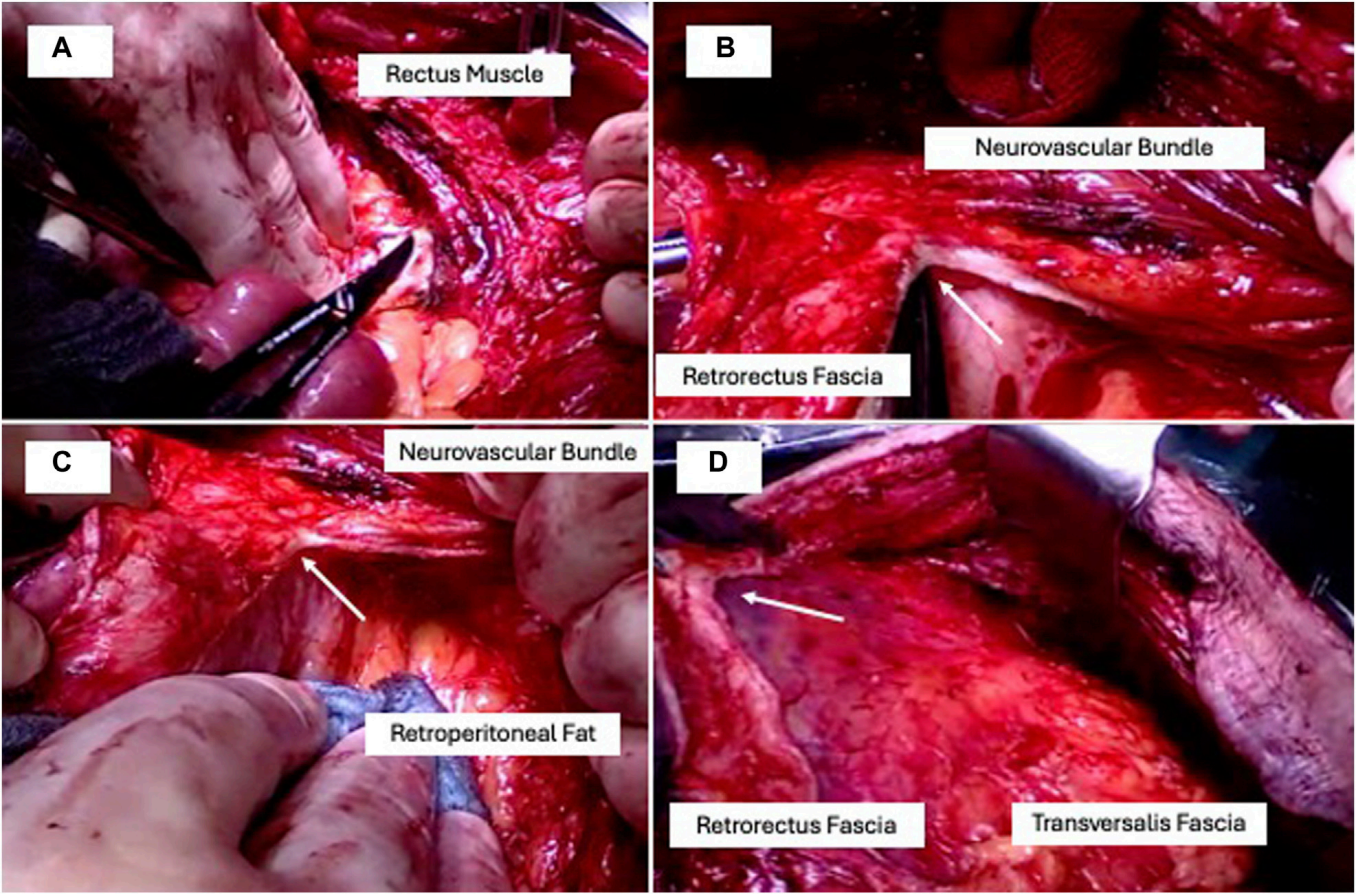
TAR in four steps: **(A)** retrorectus access, **(B)** retrorectus fascia dissection (arrow) medial to the neurovascular bundles in a bottom-up direction starting at the linea arcuata, **(C)** complete lateral mobilisation to the retroperitoneal fat, **(D)** complete cranial dissection ending dorsal to the processus xiphoideus parallel to the linea semilunaris.

At the end of this release phase an anterior muscular plane and a posterior fascial plane will have been created (Figure 1). The posterior plane is a freely mobile structure. The anterior plane merges with the hernia sac, which remains intact, and the laparotomy incision site. Maximum mobilisation of the transversalis fascia (posterior plane) is routinely achieved in all cases upon reaching the retroperitoneal fat tissue (Figure 1D).

**FIGURE 2:**
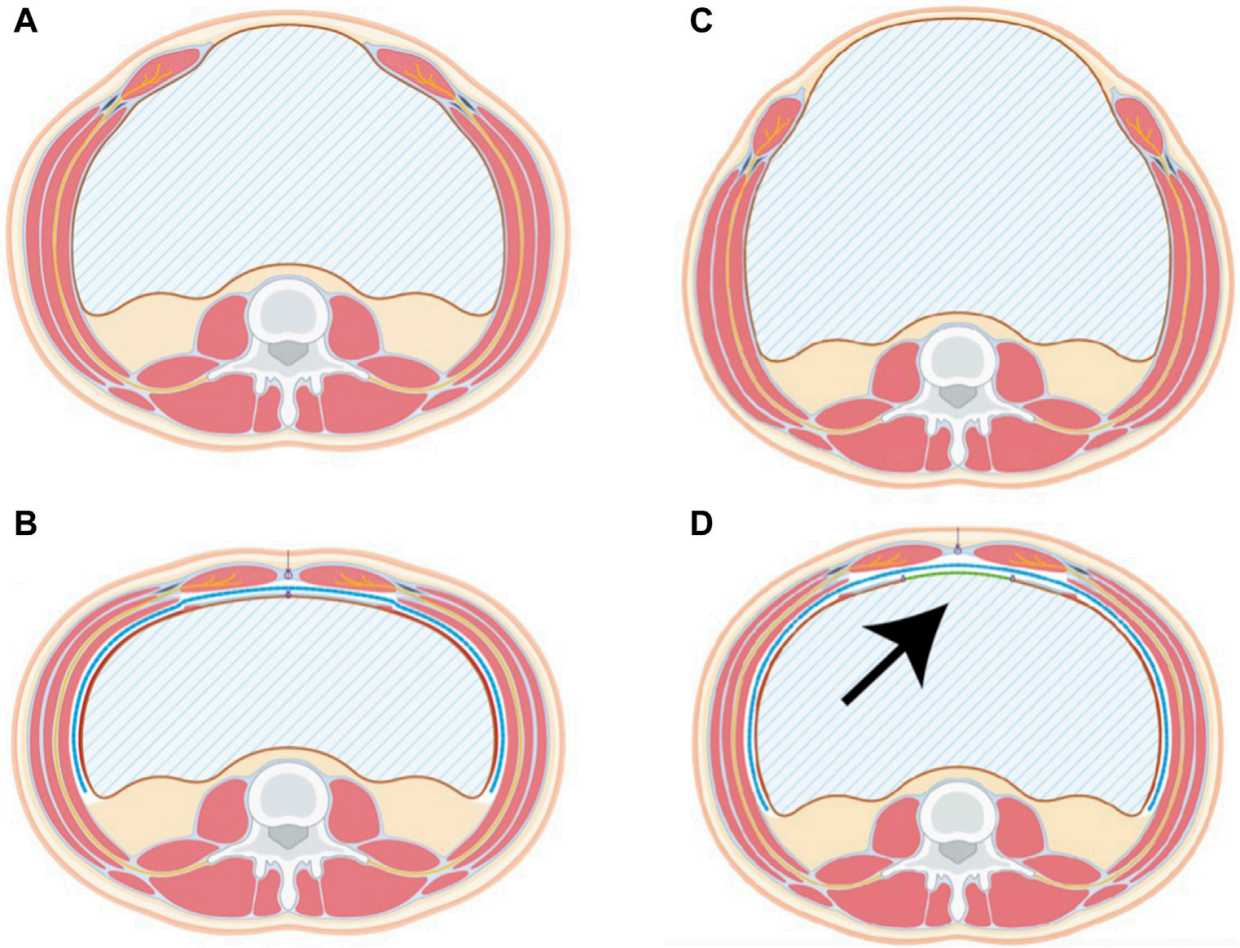
Pre- and Postoperative scheme of the TAR procedure without bridging **(A,B)** and with bridging **(C,D)**. The TAR Mesh (blue line) covers the extraperitoneal space completely while the posterior bridging mesh (arrow, green line) delimits the abdominal cavity. Remnants of the sac are used for linea alba reconstruction in both cases.

Posterior bridging is now indicated if it is not possible to close the posterior plane without tension. Posterior bridging is not an alternative to complete TAR mobilisation but our routinely performed technique in non-closable layers. Our preference here is to use a long-term absorbable mesh with an enteric adhesion barrier in an inlay technique. For this purpose, a long-term absorbable mesh made of poly-4-hydroxybutyrate with Sepra coating (Phasix ST, BD Bard, Karlsruhe, Germany) has proven its effectiveness in practice (Figure 2).

After closing the peritoneal space, the TAR mesh is placed. This should extend on both sides lateral to the retroperitoneal space and provide retroxiphoid and retro-symphyseal overlap. As standard practice, we use a Parietene macroporous mesh (Medtronic, Meerbusch, Germany) which is placed without further fixation. It may be necessary to suture together several meshes to ensure the overlapping of large defects or to fit the meshes at the edges. The TAR mesh must fully cover the newly created extraperitoneal space. Wound drains are placed on the TAR mesh and, if necessary, also at another location.

Direct closure is performed in the anterior plane with reconstruction of the linea alba. If this cannot be done while maintaining adequate strength, a non-absorbable or long-term absorbable mesh is used for prosthetic replacement of the linea alba and is sutured to the anterior fascial edge in a tension-free inlay technique. The skin is closed with skin clips, which are left in place for at least 3 weeks. An abdominal belt is applied under anesthesia. As soon as there is only a little wound drainage or the drains have been removed, we aim to discharge patients.

### Steps in Robotic TAR Repair With Additional Bridging

Patient positioning is done in physiological hyperextension. The arms are adducted and placed beside the torso. For unilateral TAR, it is helpful to position the patient close to the opposite edge of the table so that the arm can also be positioned dorsally to the body axis. This increases the mobility of the robotic arms and, in particular, facilitates ipsilateral entry into the abdominal wall. We usually place a 12 mm assist in the left or right ipsilateral upper abdomen using the open technique. The robotic trocars are then inserted along the anterior axillary line. After ventral adhesiolysis, an incision is made in the abdominal wall along the midline and this is followed by TAR preparation.

As in open surgery, we consistently divide the hernia sac from the posterior plane. It has been revealed that the hernia sac tissue cannot be developed in continuity anyway in incisional hernias and is then better integrated as a seam bearing into the anterior suture. Primary suture closure of the posterior plane inevitably results in plication of the posterior plane and thus a loss of surface area, which in small defects can be easily compensated for by the generously mobilised posterior plane. However, for large defects it is advisable to perform posterior bridging. Using this concept, the size of the extraperitoneal space is preserved and a non-absorbable TAR mesh with adequate overlap can be placed. This approach can also be applied to repair parastomal hernias. On the one hand, posterior bridging can be avoided by a more extensive or even bilateral open TAR. On the other hand, more patients may benefit from shorter operative times with minimally invasive TAR when posterior bridging is applied. After the placement of the TAR mesh, the extraperitoneal space is closed with sutures.

### Postoperative Course

Following open or robotic repair, patients are extubated and routinely monitored in the recovery room. Transfer to the intensive care unit is not routinely required. The goal is to mobilise patients from postoperative day 1 and initiate food intake. All patients receive antithrombotic medication according to the guideline recommendations.

### Perioperative Data

The procedures were performed as open and minimally invasive (conventional laparoscopic and robotic) operations. Robotic repair was carried out with a daVinci X system (Intuitive Surgical, Sunnyvale, CA, United States).

Preoperative sectional imaging was performed on all patients. Diagnostic CT imaging without contrast media is sufficient. The hernia area was calculated as an ellipse using the measured length and width (hernia area = length/2*width/2*π). The EHS classification was used to categorise hernias.

We use the term “TAR mesh” to denote a non-absorbable mesh that is placed with a wide overlap in the newly created extraperitoneal space. The area of the mesh used was calculated as a rectangle (mesh area = length*width) and the results were summed in the case of multiple meshes.

Postoperative Complications Were Graded According to the Dindo-Clavien Classification System.

Patient and procedure data were prospectively documented in the Herniamed Registry and retrospectively analysed. All patients gave informed consent for the documentation of their data in Herniamed. Statistical analysis of the 22 patients in Table 1 was performed with IBM SPSS (Version 25) using Fisher’s exact test (proportion of W3 hernias and morbidity) and non-parametric Mann Whitney U tests (age, BMI, defect width, defect length, defect size, TAR mesh size, duration of surgery, hospital stay in days).

## Results

In the 12-month period from January 01, 2023 to December 31, 2023, transversus abdominis release was performed on 50 patients as part of hernia repair. The hernias repaired were 41 ventral incisional hernias (medial: n = 27, lateral: n = 14) and 9 parastomal hernias. The median age of the patients was 64 years (24–85). The median BMI was 29 kg/m2 (21–48). Relative to the total group, morbidity was (CD1-5) 28% (n = 14). Minor complications (CD1+2) occurred in 18% (n = 9) of cases. However, surgical revision was needed in three cases (6%, CD 3b). Mortality (CD5) was 2% (n = 1) due to one patient dying of fulminant pulmonary embolism during mobilisation on postoperative day 1 following a robotic Pauli procedure.

TAR was carried out in 25 cases in open technique and in 25 cases in minimally invasive technique (robotic: n = 24, laparoscopic: n = 1). In the open surgery group, 22/25 of the hernias had a medial location. By contrast, only 5/25 of the minimally invasively repaired hernias had a medial location. The majority of the robotically repaired hernias were lateral or parastomal hernias (Table 1).

In the clinically relevant group of patients with medial hernia (n = 22) who underwent an open TAR procedure, 91% (20/22) had a W3 hernia. The median defect area in the entire group was 289 cm^2^ (31–491). None of the patients had a BMI >40 kg/m2. Based on the diagnosis and at the surgeon’s discretion, posterior bridging (n = 7) was carried out. On comparing the groups without vs. with bridging, no significant difference was seen in hernia length (=previous median laparotomy). Because of the significant difference in the defect width, there was a significant difference in the defect area. In the group of patients analysed here with bridging of the remnant posterior fascial defect, the median defect width was 24 cm. In almost all cases it was possible to reconstruct the linea alba in the anterior plane without a mesh. Only in one case was a non-absorbable mesh implanted for prosthetic replacement of the linea alba. Patients requiring additional bridging have significantly more postoperative complications. However, the length of the hospital stay is not significantly extended (Table 2).

**TABLE 2:** Perioperative outcome of open midline incisional hernia repair: ventral medial hernias with open TAR (n = 22).

	Ventral midline hernias (n = 22)		*p*
	TAR without bridging (n = 15)	TAR with bridging (n = 7)	
Age (years, Median, Min-Max)	62 (24–73)	66 (62–74)	n.s.
BMI (kg/m^2^, Median, Min-Max)	28.7 (20.6–34.7)	31.3 (24.2–39.7)	n.s.
Defect width (cm, Median, Min-Max)	15 (8–21)	24 (10–25)	0.02
Proportion of W3 hernias (>10 cm)	87% (13/15)	100% (7/7)	n.s.
Defect length (cm, Median, Min-Max)	18 (5–30)	25 (20–25)	n.s.
Defect size (cm^2^; ellipse formula: Median, Min-Max)	177 (31–396)	452 (157–491)	<0.01
Mesh bridging			
Posterior bridging		n = 7	
Additional anterior bridging		n = 1	
Size of posterior bridging mesh (cm^2^; Median, Min-Max)		600 (400–1950)	
TAR mesh size (cm^2^; rectangle formula: Median Min-Max)	1,200 (400–1750)	1750 (900–2,700)	0.05
Duration of surgery (minutes; Median, Min-Max)	139 (74–227)	222 (161–366)	<0.01
Hospital stay (days; Median, Min-Max)	6 (2–14)	10 (6–35)	n.s.
Morbidity	3/15	5/7	0.05
CD 0	n = 12	n = 2	
CD 1	n = 1	n = 1	
CD 2	n = 2	n = 2	
CD 3a	-	n = 1	
CD 3b	-	n = 1	
CD 4	-	-	
CD 5	-	-	

**FIGURE 3:**
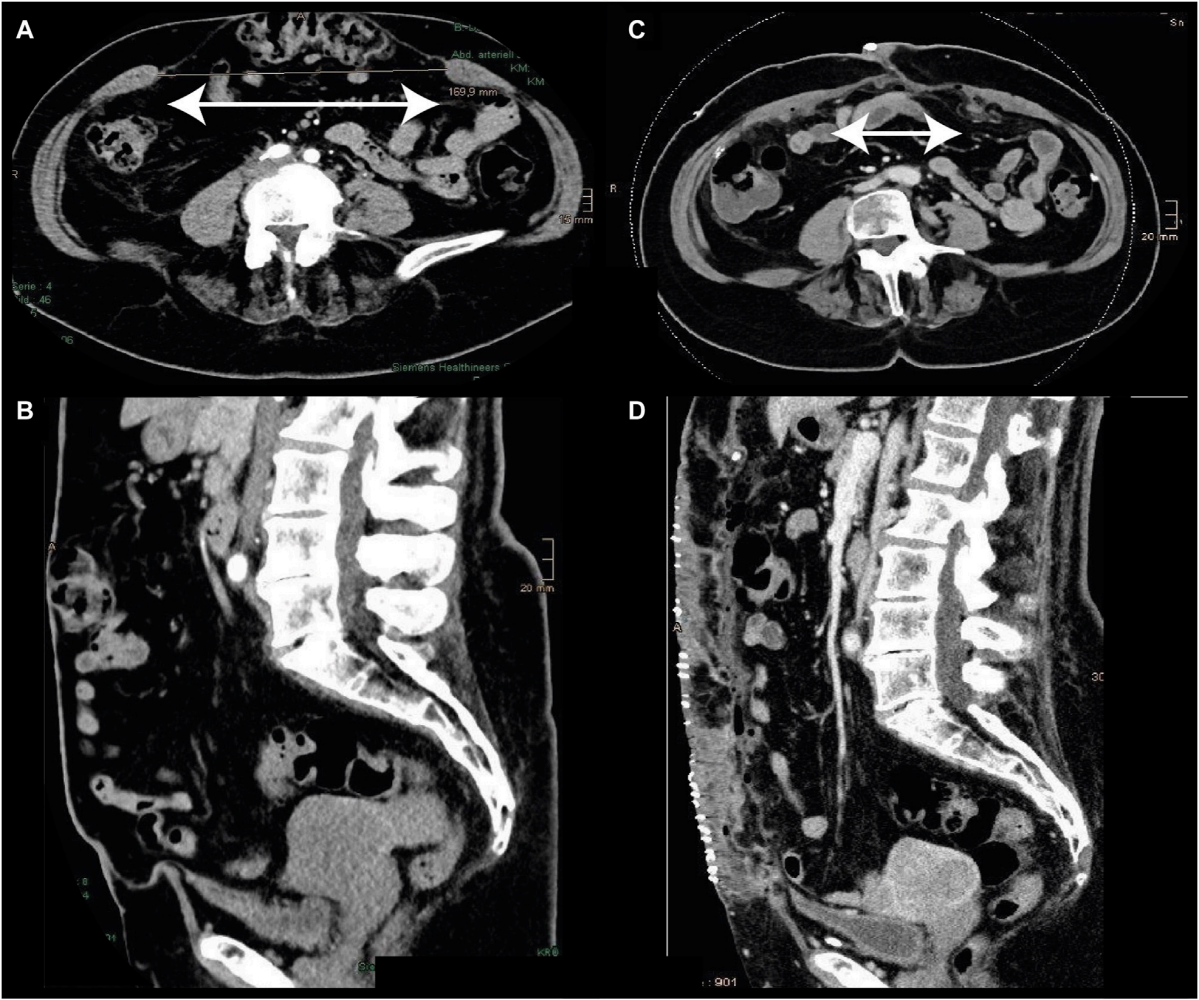
Incisional hernia [pre: **(A**,**B)**] M1-M5, W3: 30 × 17 cm. Treatment with open TAR (45 × 30 cm) and underlying posterior bridging (30 × 20 cm) mesh. The postoperative linea alba had a width of 6 cm. The duration of surgery was 189 min, with discharge from the hospital on day 4.

The standard TAR mesh used had a median size of 1,200 cm^2^. In the cases with posterior bridging (median mesh size 600 cm^2^), the median size of the TAR mesh was increased approximately by exactly this area (1750 cm^2^). For the technique presented here, the ratio of mesh size in the TAR plane to the defect size without bridging was 1,200 cm^2^/177 cm^2^ = 6.8, and with bridging it was 1750 cm^2^/452 cm^2^ = 3.8 (Figure 3). In total, posterior bridging was used in 7/22 (32%) of patients. While bilateral TAR was always performed in open medial hernia repair, bilateral TAR was performed only in 5/24 of cases in robotic TAR repair. In robotic TAR, posterior bridging was used in 3/24 (12.5%) of cases (unilateral TAR: n = 2, bilateral TAR: n = 1).

**FIGURE 4:**
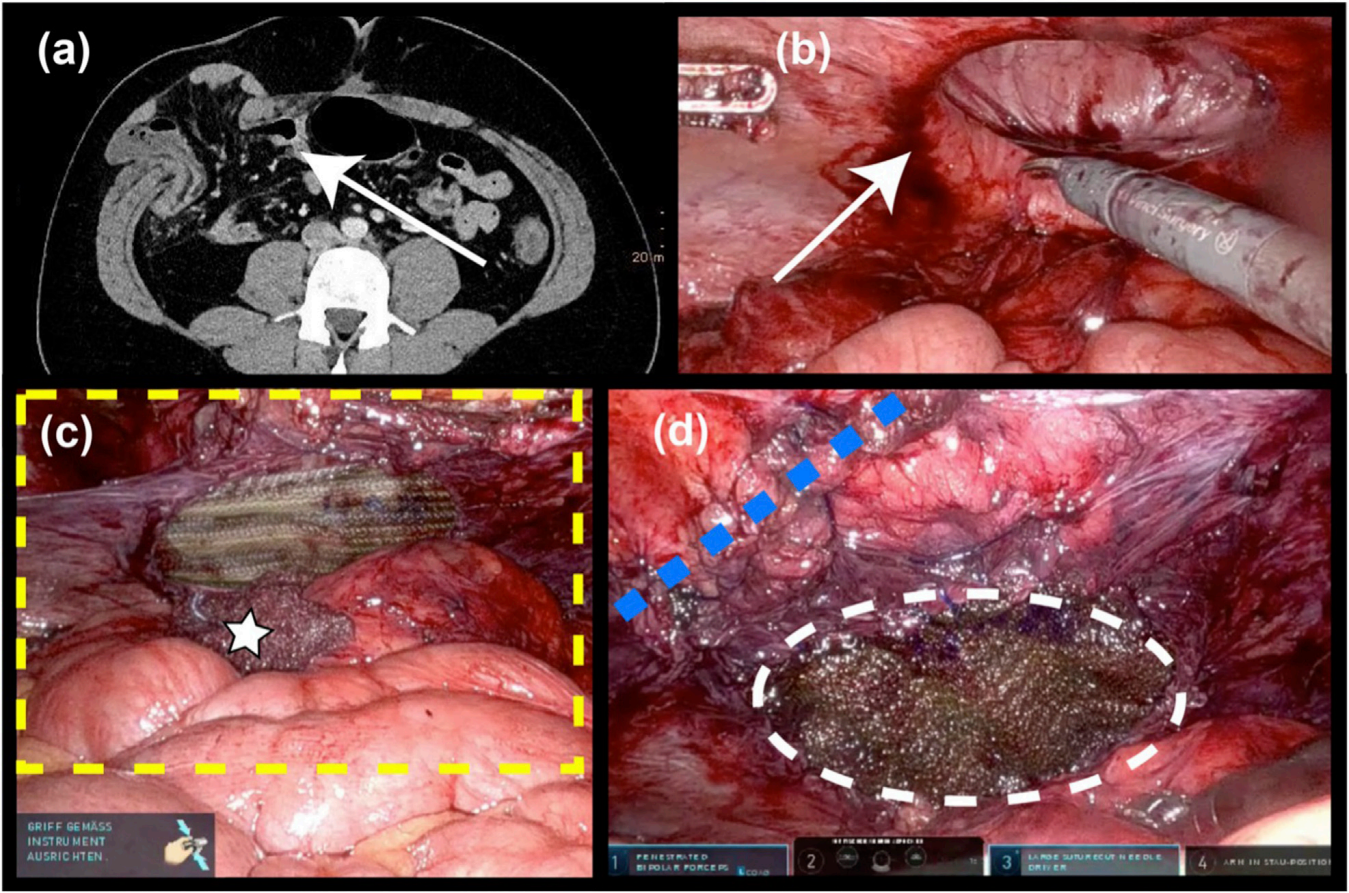
Lateral incisional hernia (6 × 6 cm). **(A)** Preop. CT scan **(B)** after robotic ventral adhesiolysis **(C)** the yellow dotted line indicates the extent of the TAR mesh (17 × 14 cm) after posterior bridging with long-term absorbable mesh (10 × 7 cm); white star: intraoperatively used swab; **(D)** the blue dotted line denotes the running suture that closes access to the extraperitoneal space; and the white dotted line marks the posterior bridging mesh.

Robotic TAR was carried out in 24 patients (Table 3). The largest group consisted of patients with lateral incisional hernia. In 7 patients with parastomal hernia the stomata were: ileal conduit: n = 5, colostoma: n = 3, enterostoma: n = 1. The hernia orifices/defects of the lateral and parastomal hernias were markedly smaller than those of the medial hernias. TAR was indicated at this location because of the anatomical boundaries of the abdominal components regardless of size. In general, unilateral docking was sufficient for lateral and parastomal hernias (18/20). The greater effort needed for the Pauli procedure was reflected in a much longer duration of surgery. Despite the smaller defects, posterior bridging was also indicated here. As can be seen in Figure 4, with this technique it was possible to preserve the extraperitoneal space for a lateral hernia without any loss of area due to plication.

**TABLE 3:** Robotic hernia surgery with TAR (n = 24), indications and outcome.

	Medial (n = 4)	Lateral (n = 13)	Pauli (n = 7)
Age	68 (60–74)	57 (27–82)	72 (57–80)
BMI	27 (24–37)	29 (21–48)	31 (22–37)
TAR bilateral	3	2	0
TAR unilateral	1	11	7
Ellipse defect size (Median, Min-Max)	47 (20–151)	16 (3–226)	13 (7–50)
Additional bridging?	1	1	1
Rectangle defect size (Median, Min-Max)	575 (216–900)	225 (100–900)	225 (196–400)
Duration of operation in minutes (Median, Min-Max)	255 (170–480)	143 (76–343)	242 (174–366)
Hospital stay in days (Median, Min-Max)	3 (2–6)	2 (2–8)	3 (1–9)
Morbidity	0/4	2/13	2/7
CD 0	n = 4	n = 11	n = 5
CD 1	-	-	n = 1
CD 2	-	n = 1	-
CD 3a	-	-	-
CD 3b	-	n = 1	-
CD 4	-	-	-
CD 5	-	-	n = 1

Subgroup analysis of medial hernias showed a pronounced procedural trend towards open TAR. The size of the defects and the number of meshes required were significantly larger. Nonetheless, the duration of surgery was shorter with open repair than with robotic repair regardless of bridging.

In the patient group analysed here, bridging was performed for very wide medial hernias to reconstruct the posterior plane. Posterior bridging was also indicated for lateral hernias in order to avoid area loss secondary to plication.

## Discussion

In addition to general optimisation of the patients (weight reduction and nicotine abstention [21]), there are individual concepts aimed at improving the local surgical conditions. Preoperative pneumoperitoneum (PPP) and preoperative Botox administration are well-known methods [22, 23]. Admittedly, PPP is restricted to specialist centres and is not yet established on a large scale. The preoperative use of Botox is much more easily reproducible. Botox is administered once and surgical repair is carried out at the time of peak effect. However, in Germany at least, it is not approved for hernia surgery. A combination of the two techniques has also been described [24, 25]. Both treatment strategies implicate the preoperative detection of patients.

We are very confident in the TAR concept and have not used any additional pre-surgical treatments like Botox or PPP. Computed tomography is mandatory to detect those patients with a potential need for bridging. Once there are intraoperative indications that a tension-free posterior closure is not possible, we use a mesh for posterior bridging. In the study period this was done in 20% (10/50) of patients and in 28% of all patients undergoing open repair of a medial hernia. The performed comparison between bridging and non-bridging patients reflects the clinical routine in our clinics and could be considered a limitation of this single centre study. Further scientific research is necessary to learn more about the outcome of bridging patients in comparison with other treatment concepts for very large hernias.

Very different approaches are described in the literature for performing posterior bridging. Some publications give the names of the mesh materials used. There are also reports of fixation of the posterior plane to the omentum, assuming it is still available to that effect. The use of the hernia sac is another option. This appears to be optimal in terms of its biological properties and the fact that it is readily available. However, we believe that the hernia sac should, always be left in place at the start of the repair so that it can be used here for the final reconstruction of the linea alba. Although the literature also reports on anterior mesh coverage with subcutaneous and connective tissue [5, 10, 26], based on our experience, the hernia sac tissue provides the most consistent stability. If the quality of the suture bed is inadequate, anterior bridging with a non-absorbable mesh may be advisable. In robotic repair of medial incisional hernias the hernia sac tissue is also left in the anterior plane and later integrated into the anterior suture. We have consistently applied this technique in open surgery.

We deliberately refrain from a stepwise forced approximation of the left and right rectus fascia, attempting instead to achieve the physiological reconstruction of the linea alba. From clinical observation, we are not aware of any disadvantages. So far, we do not have follow-up data on our patients. To our knowledge there are no published studies reporting on the ideal width of a reconstructed linea alba in relation to TAR repair. The low rate of patients with severe complications in the group reported here appears to confirm the benefits of the described procedure.

A much-feared and life-threatening complication of hernia surgery is abdominal compartment syndrome [27–30]. It is caused by an imbalance between the space needed by the abdominal organs and the space available in the newly created abdominal cavity. Previous surgical concepts aimed at countering this included, apart from omentum resection, even bowel resection. The focus here is on abdominal wall-related treatment concepts. The most widely established of these is the anterior [31] and posterior [1] component separation. A newer method is biochemical component separation with Botox, possibly in combination with progressive pneumoperitoneum [32, 33]. The fasciotens system (Dahlhausen, Cologne, Germany) is another innovative method designed for midline reconstruction through intraoperative traction [34]. We believe that tension-free reconstruction and extraperitoneal placement of a non-absorbable mesh with sufficient overlap are the cornerstones of optimal hernia repair [8]. With the technique presented here, the TAR mesh covers the defect by a factor of 6.8 in cases without bridging and by a factor of 3.8 in cases with bridging.

Reconstruction of the linea alba may take place as a result of impaired wound healing. For this reason, further medialisation of the anterior plane could make sense. A combination of anterior and posterior component separation would represent a major step forward [20]. We believe that the associated weakening of the lateral abdominal wall is a disadvantage that has not been properly investigated so far. With this in mind, we feel that accepting a mesh-reinforced rectus diastasis is the best compromise if low-risk reconstruction is attempted. Further studies should be carried out to investigate the functional outcomes of patients in the long term. Perioperative observations do not point to any abnormalities. Another option could be the preoperative application of Botox to the anterior muscle layer. This would theoretically facilitate the reconstruction of the linea alba. However, we do not expect any effect on the need for posterior bridging in our technique as the medial extent of the posterior layer is mainly influenced by the preservation of the hernia sac in the anterior layer.

For lateral and parastomal hernias unilateral TAR is generally sufficient to generate an extraperitoneal space with adequate mesh overlap. As mentioned above, a defect in the size of the hernia orifice may occur in the posterior plane. Bridging in this setting helps to preserve the extraperitoneal mesh bed (landing zone) as necessary for the TAR mesh. A possible technical alternative would be the use of coated meshes. However, based on our observations, it is easier to begin with the reconstruction of the posterior plane and then focus on the optimal placement of the TAR mesh. In the study period presented here, there were no patients with loss of domain at this location. We therefore did not include theoretical considerations in this publication.

## Summary

TAR is an important surgical tool in the repair of ventral and parastomal hernias. It offers unique opportunities through the tension-free placement of meshes with adequate overlap. Posterior bridging is a useful adjunct that can be used here depending on the intraoperative findings. There is no need for the preoperative selection of patients for special pretreatment. Therefore, this concept offers very high flexibility in the routine treatment of these patients.

## Data Availability

The raw data supporting the conclusion of this article will be made available by the authors, without undue reservation.

## References

[1] Novitsky YW Elliott HL Orenstein SB Rosen MJ. Transversus Abdominis Muscle Release: A Novel Approach to Posterior Component Separation During Complex Abdominal Wall Reconstruction. Am J Surg (2012) 2045:709–16. 10.1016/j.amjsurg.2012.02.008 22607741

[2] Pauli EM Juza RM Winder JS. How I Do it: Novel Parastomal Herniorrhaphy Utilizing Transversus Abdominis Release. Hernia (2016) 204:547–52. 10.1007/s10029-016-1489-3 27023876

[3] Wegdam JA Thoolen JMM Nienhuijs SW de Bouvy N de Vries Reilingh TS. Systematic Review of Transversus Abdominis Release in Complex Abdominal Wall Reconstruction. Hernia (2019) 231:5–15. 10.1007/s10029-018-1870-5 30539311

[4] Zolin SJ Fafaj A Krpata DM. Transversus Abdominis Release (TAR): What Are the Real Indications and Where Is the Limit? Hernia (2020) 242:333–40. 10.1007/s10029-020-02150-5 32152808

[5] Novitsky YW Fayezizadeh M Majumder A Neupane R Elliott HL Orenstein SB. Outcomes of Posterior Component Separation With Transversus Abdominis Muscle Release and Synthetic Mesh Sublay Reinforcement. Ann Surg (2016) 2642:226–32. 10.1097/SLA.0000000000001673 26910200

[6] Hodgkinson JD Leo CA Maeda Y Bassett P Oke SM Vaizey CJ A Meta-Analysis Comparing Open Anterior Component Separation With Posterior Component Separation and Transversus Abdominis Release in the Repair of Midline Ventral Hernias. Hernia (2018) 224:617–26. 10.1007/s10029-018-1757-5 29516294

[7] Oprea V Mardale S Buia F Gheorghescu D Nica R Zdroba S The Influence of Transversus Abdominis Muscle Release (TAR) for Complex Incisional Hernia Repair on the Intraabdominal Pressure and Pulmonary Function. Hernia (2021) 25:1601–9. 10.1007/s10029-021-02395-8 33751278 PMC7983096

[8] Jones CM Winder JS Potochny JD Pauli EM. Posterior Component Separation With Transversus Abdominis Release: Technique, Utility, and Outcomes in Complex Abdominal Wall Reconstruction. Plast Reconstr Surg (2016) 1372:636–46. 10.1097/01.prs.0000475778.45783.e2 26818302

[9] San Miguel-Méndez C López-Monclús J Munoz-Rodriguez J de Lersundi ÁRV Artes-Caselles M Blázquez Hernando LA Stepwise Transversus Abdominis Muscle Release for the Treatment of Complex Bilateral Subcostal Incisional Hernias. Surgery (2021) 1704:1112–9. 10.1016/j.surg.2021.04.007 34020792

[10] Alkhatib H Tastaldi L Krpata DM Petro CC Olson M Rosenblatt S Outcomes of Transversus Abdominis Release in Non-Elective Incisional Hernia Repair: A Retrospective Review of the Americas Hernia Society Quality Collaborative (AHSQC). Hernia (2019) 231:43–9. 10.1007/s10029-019-01878-z 30627813

[11] Carbonell AM Cobb WS Chen SM. Posterior Components Separation During Retromuscular Hernia Repair. Hernia (2008) 124:359–62. 10.1007/s10029-008-0356-2 18293053

[12] Krpata DM Blatnik JA Novitsky YW Rosen MJ. Posterior and Open Anterior Components Separations: A Comparative Analysis. Am J Surg (2012) 2033:318–22. discussion 322. 10.1016/j.amjsurg.2011.10.009 22244073

[13] Kushner B Holden S Blatnik J. Surgical "Error Traps" of Open Posterior Component Separation-Transversus Abdominis Release. Hernia (2021) 256:1703–14. 10.1007/s10029-020-02321-4 33079331

[14] Punjani R Arora E Mankeshwar R Gala J. An Early Experience With Transversus Abdominis Release for Complex Ventral Hernias: A Retrospective Review of 100 Cases. Hernia (2021) 252:353–64. 10.1007/s10029-020-02202-w 32377962

[15] Robin-Lersundi A Blazquez Hernando L López-Monclús J Cruz Cidoncha A San Miguel Méndez C Jimenez Cubedo E How We Do it: Down to up Posterior Components Separation. Langenbecks Arch Surg (2018) 4034:539–46. 10.1007/s00423-018-1655-4 29502282

[16] Siegal SR Pauli E. Posterior Component Separation/Transversus Abdominis Release. Plast Aesthet Res (2019) 6:25. 10.20517/2347-9264.2019.35

[17] Winder JS Majumder A Fayezizadeh M Novitsky YW Pauli EM. Outcomes of Utilizing Absorbable Mesh as an Adjunct to Posterior Sheath Closure During Complex Posterior Component Separation. Hernia (2018) 222:303–9. 10.1007/s10029-018-1732-1 29349616

[18] Zolin SJ Krpata DM Petro CC Prabhu AS Rosenblatt S Rosen S Long-Term Clinical and Patient-Reported Outcomes After Transversus Abdominis Release With Permanent Synthetic Mesh: A Single Center Analysis of 1203 Patients. Ann Surg (2023) 2774:e900–e906. 10.1097/sla.0000000000005443 35793810

[19] Riediger H Kockerling F. Open Transversus Abdominis Release in Incisional Hernia Repair: Technical Limits and Solutions. Hernia (2024). 10.1007/s10029-024-02994-1 38548919

[20] Sneiders D de Smet GHJ den Hartog F Verstoep L Menon AG Muysoms FE Medialization After Combined Anterior and Posterior Component Separation in Giant Incisional Hernia Surgery, an Anatomical Study. Surgery (2021) 1706:1749–57. 10.1016/j.surg.2021.06.018 34417026

[21] Knaapen L Buyne O Slater N Matthews B Goor H Rosman C. Management of Complex Ventral Hernias: Results of an International Survey. BJS Open (2021) 5:zraa057. 10.1093/bjsopen/zraa057 33609388 PMC7893472

[22] Barretto VRD de Oliveira JGR Brim ACS Araujo RBS Barros RA Romeo ALB. Botulinum Toxin A in Complex Incisional Hernia Repair: A Systematic Review. Hernia (2023). 10.1007/s10029-023-02892-y 37801164

[23] Renard Y Lardiere-Deguelte S de Mestier L Appere F Colosio A Kianmanesh R Management of Large Incisional Hernias With Loss of Domain: A Prospective Series of Patients Prepared by Progressive Preoperative Pneumoperitoneum. Surgery (2016) 1602:426–35. 10.1016/j.surg.2016.03.033 27262533

[24] Bueno-Lledo J Carreno-Saenz O Torregrosa-Gallud A Pous-Serrano S. Preoperative Botulinum Toxin and Progressive Pneumoperitoneum in Loss of Domain Hernias-Our First 100 Cases. Front Surg (2020) 7:3. 10.3389/fsurg.2020.00003 32181259 PMC7059432

[25] Tashkandi A Bueno-Lledó J Durtette-Guzylack J Cayeux A Bukhari R Rhaeim R Adjunct Botox to Preoperative Progressive Pneumoperitoneum for Incisional Hernia With Loss of Domain: No Additional Effect but May Improve Outcomes. Hernia (2021) 256:1507–17. 10.1007/s10029-021-02387-8 33686553

[26] Garcia-Urena MA Lopez-Monclus J Cuccurullo D Blazquez Hernando LA Garcia-Pastor P Reggio S Abdominal Wall Reconstruction Utilizing the Combination of Absorbable and Permanent Mesh in a Retromuscular Position: A Multicenter Prospective Study. World J Surg (2019) 431:149–58. 10.1007/s00268-018-4765-9 30132226

[27] Cruz GV Méndez García C Gómez Benítez G Ramos Domínguez V. Giant Loss of Domain Hernia: Preoperative Treatment With Botulinum Toxin of the Abdominal Oblique Muscles. Rehabilitacion (Madr) (2019) 534:284–7. 10.1016/j.rh.2018.12.003 31813424

[28] Elstner KE Read JW Rodriguez-Acevedo O Ho-Shon K Magnussen J Ibrahim N. Preoperative Progressive Pneumoperitoneum Complementing Chemical Component Relaxation in Complex Ventral Hernia Repair. Surg Endosc (2017) 314:1914–22. 10.1007/s00464-016-5194-1 27572061

[29] Hever P Dhar M Cavale N. Loss of Domain Leading to Intra-Operative Cardiorespiratory Arrest During Open Repair of a Giant Inguinoscrotal Hernia and Hydrocele. JPRAS Open (2018) 16:1–5. 10.1016/j.jpra.2017.11.005 32158804 PMC7061612

[30] Tang FX Ma N Xie XX Chen S Zong Z Zhou TC. Preoperative Progressive Pneumoperitoneum and Botulinum Toxin Type A in Patients With Large Parastomal Hernia. Front Surg (2021) 8:683612. 10.3389/fsurg.2021.683612 34164428 PMC8215116

[31] Ramirez OM Ruas E Dellon AL. Components Separation" Method for Closure of Abdominal-Wall Defects: An Anatomic and Clinical Study. Plast Reconstr Surg (1990) 863:519–26. 10.1097/00006534-199009000-00023 2143588

[32] de Jong DLC Wegdam JA Van der Wolk S Nienhuijs SW de Vries Reilingh TS. Prevention of Component Separation in Complex Abdominal Wall Surgery by Botox Prehabilitation: A Propensity-Matched Study. Hernia (2024). 10.1007/s10029-023-02929-2 38172376

[33] Dias ERM Rondini GZ Amaral PHF Macret JZ Carvalho JPV Pivetta LGA Systematic Review and Meta-Analysis of the Pre-Operative Application of Botulinum Toxin for Ventral Hernia Repair. Hernia (2023) 274:807–18. 10.1007/s10029-023-02816-w 37329437

[34] Niebuhr H Malaibari ZO Kockerling F Reinpold W Dag H Eucker D Intraoperative Fascial Traction (IFT) for Treatment of Large Ventral Hernias: A Retrospective Analysis of 50 Cases. Chirurg (2022) 933:292–8. 10.1007/s00104-021-01552-0 PMC8894171 34907456

